# Hemicellulose from cane bagasse as assessed by OLIMP MALDI-TOF/MS

**DOI:** 10.1186/1753-6561-8-S4-P104

**Published:** 2014-10-01

**Authors:** Francis Lopes, Márcio Barbosa, Marcelo Loureiro

**Affiliations:** 1 Universidade Federal de Goiás, Goiânia, Brazil; 2 Universidade Federal de Viçosa, Viçosa, Brazil

## 

Hemicelluloses are abundant polysaccharides in the plant cell walls. They tether the celulose microfibrils and participate in the control of the cell expansion together with expansins (Cosgrove, 1998) [1]. The hemicellulose may present a variety of decorating side chains, including feruloyl and acetyl substituents. The ferulic acid may be esterified to the hemicellulose and cross-linked to lignin, tethering lignin and hemicellulose to each other, increasing both hydrophobicity and recalcitrance to enzymatic digestions.

The establishment of easy accession methods to investigate the structure of the complex lignin-carbohydrate (CLC) speed up the investigation of naturally occurring or genetically introduced biomass features. The oligosaccharide mass profiling (OLIMP) consists in the cell wall digestion with specific fungal enzymes followed by the assessment of the oligosaccharides released by MALDI-TOF/MS (Wang et al., 1999). It is simple, informative and fast, allowing for depicting the closer native structure of polymers in muro. This work aimed to assess the hemicellulose structures occurring in bagasse of different varieties of canes by OLIMP MALDI-TOF/MS.

The oligo structures were investigated in two cell wall fractions. Cell wall residue from bagasse (BCWR) consisted in the trituration of bagasse with liquid N_2 _followed by sequential extractions in 80% ethanol and chloroform:methanol (1:1 v/v). The alkali extracted hemicellulose (AEH) came from serial alkali extraction of extractive free bagasse powder in 0.1M, 1.0M and 4.0M NAOH. The oligo structures were released upon digestion of BCWR or AEH with Pentopan Mono BG^® ^or Driselase (Sigma) in 0,1M sodium acetate buffer, pH 5,6; at 37°C or 42°C overnight, respectively. The supernatant of the digestions were co-crystalized with 20 mg/ml dihydroxybenzoic acid, prepared in 70% acetonitrile/0,1% trifluoroacetic acid. The peaks were collected in a Brucker Daltonics equipment and analyzed with the Flex Analysis 3.0 software.

Oligosaccharides with m/z 701 and 833 predominated the spectra from AEH/Pentopan Mono BG^® ^for all varieties of sugarcane studied. The peak of m/z 701 was confirmed to be a xylopentaose (Xyl5) in MS2 mode. The peak of m/z 833,3 may represent a hexa-oligosaccharide (Sorensen et al., 2007) [2].

BCWR/Pentopan Mono BG^® ^allowed for the detection of many feruloylated oligosaccharides. In addition, the analysis of BCWR/Driselase allowed for the detection of arabino-xylo, feruloyl-arabino-xylo and acetylated feruloyl-arabino-xylo oligosaccharides. In this experimental, the oligosaccharide of m/z 745,2 was abundant and possibly represents a feruloylated tetra-oligosaccharide. The RB867515 and *Saccharum sinensis *BCWR/Driselase digestions exhibited abundance of the oligo structure with m/z 613, which may correspond to a feruloylated trisaccharide (Xyl2AraFerA). Acetyl substitutions (increases of 42 Da) upon the oligo structures of m/z 613, 745 and 2507,8 may have rendered the m/z shifts of 655, 787 and 2549,9 detected.

The xylopentaose (Xyl5) predominated the oligosaccharide profiles for AEH digested with Pentopan Mono BG^® ^for all varieties of sugarcane studied, suggesting that the synthesis of xylan in *Saccharum *may conserve a core pentasaccharide structure (Xyl5) eventually modified with acetyl and feruloyl substituents.
